# An Outline of Medical Chemistry for the Use of Students

**Published:** 1855-08

**Authors:** 


Art. VI.—*An Outline of Medical Chemistry for the use of Students.* By B. Howard Rand, A. M., M. D., Professor of Chemistry in the Philadelphia College of Medicine; Lecturer on General Chemistry to the Franklin Institute of the State of Pa.; Recording Secretary of the Academy of Natural Sciences of Philadelphia; Fellow of the Philadelphia College of Physicians. Philadelphia: Lindsay & Blakiston, 1855; pp. 259, 24mo.

This little work exhibits the science of Chemistry in the utmost state of compression. It is a summary of the principal facts pertaining to that interesting branch of study, designed to refresh the memory of the student. All such aids as it purports to be are required by the student of medicine, who, at most, can hope during his brief pupilage to acquire only a general knowledge of chemistry. This science has grown to be so extensive and so intricate that its mastery demands the undivided attention of the student. The medical man cannot give this, and must be contented with the essentials of the science. We commend to students this syllabus of Dr. Rand.

